# The palliative care needs and experiences of patients with advanced Parkinson’s disease: a qualitative scoping review

**DOI:** 10.3389/fmed.2024.1362828

**Published:** 2024-04-10

**Authors:** Yan Lou, Yiting Li, Yiping Chen

**Affiliations:** ^1^School of Nursing, Hangzhou Normal University, Hangzhou, Zhejiang, China; ^2^Zhejiang Haining Health School, Haining City, Zhejiang, China; ^3^School of Nursing, Shanxi Medical University, Taiyuan, Shanxi, China

**Keywords:** palliative care, Parkinson’s disease, needs, experiences, scoping review

## Abstract

**Aim:**

To determine the experiences and needs of palliative care in patients with advanced Parkinson’s disease (PD).

**Methods:**

A scoping literature review methodology, as described by the Joanna Briggs Institute, was employed to search for relevant literature. An electronic search of studies published in English was conducted across five databases from inception to 10 September 2023.

**Results:**

The search yielded a total of 1,205 articles, with 20 meeting the inclusion criteria. The findings were organized into four themes: (1) unmet emotional and informational needs; (2) needs for effective coordination of care; (3) planning for the future; and (4) symptom management. This scoping review highlights the intricate nature of palliative care for patients with PD and sheds light on issues within current palliative care healthcare systems. The findings emphasize the necessity for individualized interventions and services to address the diverse unmet palliative care needs of people with PD.

**Conclusion:**

The study reveals the complex landscape of palliative care for individuals with advanced PD, emphasizing the inadequacies within existing healthcare systems. The identified themes underscore the importance of tailored interventions to address the varied unmet palliative care needs of this population.

## 1 Introduction

Parkinson’s disease (PD) is a progressive neurodegenerative disease characterized by motor and non-motor symptoms (1). In 2016, it was estimated that PD affected 6.1 million people worldwide, and it is expected that the rate of growth in numbers of patients will be the highest of all neurological disease, even that of Alzheimer’s disease (2). The progression of PD can be described as experiencing a phase trajectory, starting from the “honeymoon” phase where symptoms are almost entirely alleviated due to medication, followed by the motor complications phase, the neuropsychiatric phase, and finally entering the palliative phase (3). The treatment for early-stage PD is entirely different from that of the advanced stage. Early-stage PD treatment requires minimizing motor disturbances and reducing the occurrence of both motor and non-motor off times to maximize independent motor function. However, in the advanced stage, patients with advanced PD have escalating disabilities and a rising number of symptoms, there are almost no available treatment options, and the focus of treatment shifts to addressing primary non-motor symptoms with more supportive and palliative measures (4). Therefore, palliative care is especially important for patients with advanced Parkinson’s disease.

According to the WHO definition (5), palliative care is an approach aimed at enhancing the wellbeing of patients and their families when dealing with challenges related to serious illnesses. This involves early identification, thorough assessment, and effective management of pain and other concerns, whether they are physical, emotional, or spiritual. Advanced PD presents a significant symptom burden, encompassing pain, fatigue, daytime drowsiness, mobility issues, depression, cognitive impaired, and so on, which requiring timely planning and potential end-of-life considerations (6). Moreover, advanced PD imposes a substantial strain on caregivers, making their caregiver roles challenging. A meta-ethnography highlighted the complex and dynamic experiences of family caregivers for PD patients, underlining the heavy burden associated with caring for those with PD (7).

There is evidence showing that palliative care specialists in multidisciplinary PD clinics can improve patients’ and caregivers’ quality of life as well as other outcomes (8). Palliative care has been demonstrated to improve self-perception and minimize stress, anger, and loss of control (9, 10). Additionally, it helped patients with their disease burden (11). Despite these potential benefits, there is still confusion about the role of palliative care in PD progression, and palliative care participation is likely being underutilized. PD patients are less likely to obtain palliative care services than patients with other neurological disorders, a study found that hospice deaths were exceedingly rare in patients with PD, occurring in just 0.6% of this population (12). Patients with advanced PD in Germany have a drastically deteriorated quality of life, with 72% having an unmet need for palliative care and only 2.6% having contact with palliative care providers (13).

As the potential benefits of palliative care for advanced PD are gradually acknowledged, an increasing number of researchers are turning to qualitative research methods to explore the specific needs and experiences of this group regarding palliative care. Compared to quantitative research, qualitative studies provide a deeper understanding, helping researchers interpret the complexity, depth, and meaning of certain phenomena (14). However, there currently exists no literature review specifically focused on qualitative research concerning the palliative care needs and experiences of patients with advanced PD. A scoping review can provide comprehensive insights and is crucial in showcasing the types of evidence that can inform practice and identify significant areas lacking evidence (15). Therefore, through this qualitative scoping review, our aim is to comprehensively understand and identify the palliative care needs and experiences of patients with advanced PD and their caregivers. By doing so, we aspire to make significant contributions to the development of pertinent care models and to pinpoint areas for future investigation.

## 2 Methods

The five-stage scoping review framework proposed by Arksey and O’Malley (16) was employed. The goal was to compile a list of advanced PD patients’ palliative care needs and experiences. Because of the scoping review’s nature, no attempt was made to assess the quality of the papers included. Instead, the review was guided by the five-stage methodology shown below.

### 2.1 Determining the study question

This stage entails determining the research question as well as this review’s principal goal. The primary goal of this study was to compile current evidence on the needs and experiences of patients with advanced PD in palliative care from the perspectives of patients and their caregivers. The research question was: What are the needs and experiences of patients with advanced PD and their caregivers in terms of palliative care?

### 2.2 Identifying relevant studies

The literature was thoroughly assessed in the following five databases from the inception (1 January 1952) to 10 September 2023: PubMed, Embase, EBSCOhost CINAHL, ProQuest, and Scopus. Two members of the research team designed a keyword-based search strategy, which was approved by the third member of the research team. We are using the key words Parkinson’s disease, palliative care, qualitative research identified as relevant Medical Subject Headings (MeSH). Free text words added to the search were supportive care, qualitative, need*, experience*, and so on, see Supplementary File 1. The different keywords were combined using Boolean operators, such as AND or OR. Thesaurus phrases were chosen to complement the keywords based on an individual database. All of the included articles’ reference lists were also meticulously searched for any other papers that discussed palliative care experiences and requirements.

### 2.3 Study selection

The literature period was from 1 January 1952 to 10 September 2023, to assure the theme’s comprehensiveness. For the selection of studies, the following inclusion criteria were considered: (a) original qualitative research articles which detailed participants’ direct reports of their supportive and palliative care needs; (b) made available in the full version; and (c) English languages. Exclusion criteria were defined: (a) a mixed population and not focus on advanced PD separately; (b) the sample of advanced PD less than 50%; (c) the focus of the article was medical/clinical treatment, deep brain stimulation, or prognostication or reporting unmet supportive care needs specific to the COVID-19 pandemic (outside of scope); (d) focusing on a single aspect of palliative care services (e.g., spiritual care only); and (e) conferences, public policies, and videos. The scoping review’s inclusion and exclusion criteria were determined after obtaining knowledge with the existing literature on palliative care. The first phase of screening looked at the titles and abstracts to see if they met the inclusion and exclusion criteria. The entire texts of selected papers from the first round of screening were assessed in the second step. Any discrepancies between the two reviewers were resolved by a two-reviewer consensus or the consultation of a third reviewer to confirm the final inclusion.

### 2.4 Charting the data

Depending on the research questions, the following essential pieces of data from the included publications were compiled in an iteratively designed data chart form: first author, country, aims, setting, participants, methods, and findings. This chart form was used to compile data from all of the listed studies in a narrative format.

### 2.5 Compiling, analyzing, and reporting the findings

We used six steps of thematic analysis (17) to define the needs: (1) Familiarization with the data: two researchers repeatedly read the included literature to become thoroughly acquainted with it. (2) Generating initial codes: once familiar with the data, the next step involves coding the data. In our research, this was done independently by the two team members. Coding involves highlighting words, phrases, or sections of the data that appear significant and tagging them with a descriptive label. (3) Searching for themes: after coding, the next step is to collate codes into potential themes. In our research, this involved an iterative process where coding results were continuously revised until the themes comprehensively covered all needs and experiences mentioned in the literature. (4) Reviewing themes: this involves refining and defining the themes to ensure they have a clear scope and focus. To ensure consistency and validate the process, two reviewers conducted a thematic analysis separately. (5) Defining and naming themes: once the themes are refined, they are clearly defined and named. The results were segmented into literature aspects and subthemes that address PD patients’ palliative care experiences and needs. (6) Producing the report: after performing critical comparisons and debates, the research team unanimously supported the findings.

## 3 Results

**FIGURE 1:**
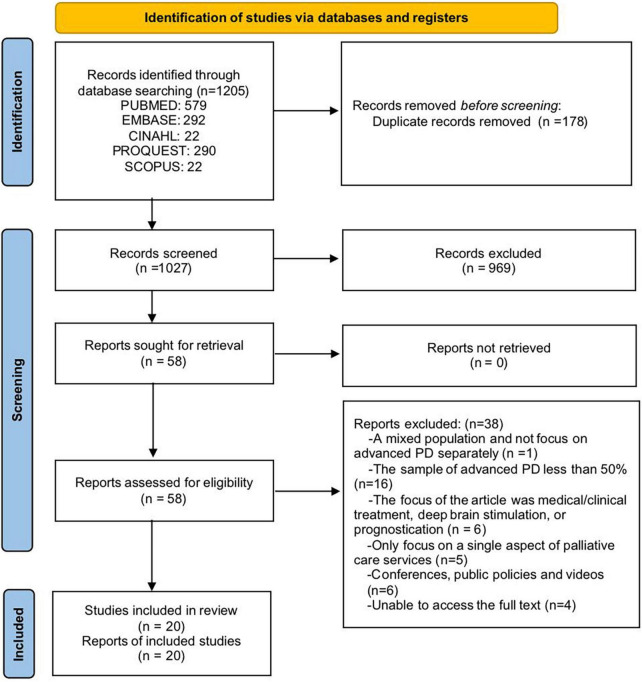
PRISMA diagram of the literature search. Adapted from Page et al. (58).

Our search yielded 1,206 articles from the five databases combined. After excluding duplicates (*n* = 178), the titles and abstracts of the remaining 1,027 studies were screened, and 58 articles were subjected to full-text reviews. Based on the inclusion and exclusion criteria, 20 studies were identified as eligible for this study. A flowchart of the search strategy and selection procedure is shown in Figure 1.

### 3.1 Aspects of the literature

The general elements of the reviewed literature are presented in Table 1. Most of the 20 studies included were performed in the United Kingdom (*n* = 7), and the remaining were from the United States (*n* = 3), Canada (*n* = 3), Netherlands (*n* = 1), Italy (*n* = 1), Australia (*n* = 1), Dutch (*n* = 1), Singapore (*n* = 1), Sweden (*n* = 1), and China (*n* = 1). There were 533 participants (238 patients, 254 caregivers, and 41 healthcare professionals). Among these 20 papers, 6 focused solely on the perspectives of PD patients, 5 focused solely on the perspectives of PD caregivers, 7 presented information on the patients and caregivers, and the other paper on the patients, caregivers, and healthcare professionals. The precise elements of the papers included are described in Tables 1, 2.

**TABLE 1:** Characteristics of included studies (*N* = 20).

References	Country	Aim	Setting	Participants
1. Hudson et al. (18)	Australia	The purpose of this project was to describe the experience of PD and consider the relevance of palliative care for this population	Three Australian states (Western Australia, Queensland, and Victoria)	PD patients, *N* = 8; caregivers, *N* = 21; professionals, *N* = 6
2. Giles and Miyasaki (19)	Canada	To understand participant’s lived healthcare experiences and the needs flowing from them	A Canadian tertiary care academic teaching hospital that did not have a multidisciplinary team approach to patient care	PD patients, *N* = 3; caregivers, *N* = 4
3. Hasson et al. (20)	United Kingdom	To explore former carers’ lived experiences of palliative and end-of-life care	In GP surgeries and libraries and Parkinson’s disease support group meetings	Caregivers, *N* = 11
4. Tan et al. (21)	Singapore	To conduct an in-depth qualitative examination of the experiences of Singaporean people caring for those with PD	The Singapore General Hospital neurology specialist outpatient clinic	Caregivers, *N* = 21
5. Beaudet and Ducharme (22)	Canada	To identify the principal intervention needs of elderly couples living with moderate-stage Parkinson’s disease and their preferences regarding the modalities of a possible nursing intervention	An outpatient neurology clinic of a hospital in the Montreal region	PD patients, *N* = 10; caregivers, *N* = 10
6. Seamark et al. (23)	England	To elicit family carers’ views about the community support that made death at home possible	At the participants’ homes	Caregivers, *N* = 59
7. van Rumund et al. (24)	Netherlands	To analyze the quality of PD care in Dutch nursing homes from the perspective of residents, caregivers, and healthcare workers	Twelve large nursing home organizations, situated in southeast Netherlands	PD patients, *N* = 15; caregivers, *N* = 15; nurses, *N* = 13; nursing home professionals, *N* = 22
8. Murdock et al. (25)	United Kingdom	To explore how people with advanced PD experience the phenomenon of occupation in their daily lives in order to inform the practice of occupational therapy in palliative care	Seven interviews were conducted in a participant’s own home and three in suitable venues at their convenience	PD patients, *N* = 11
9. Fox et al. (26)	United Kingdom	To explore the palliative care and related issues most affecting people with PD and their families and to examine perceptions about/understanding of palliative care	Three movement disorder clinics in Cork, Ireland	PD patients, *N* = 19; caregivers, *N* = 12
10. Habermann and Shin (27)	United States	To explore how couples with PD discuss their needs, concerns, and preferences at the advanced stages of illness	A movement disorder practice and from Parkinson’s support groups in the Midwest region of the United States	PD patients, *N* = 14; caregivers, *N* = 14
11. Hurt et al. (28)	United Kingdom	To investigate the nature of illness uncertainty in the carers of patients with PD	Conducted over the telephone	Caregivers, *N* = 18
12. Hurt et al. (28)	United Kingdom	To explore barriers to help-seeking using two theoretical frameworks	At King’s College Hospital and Lewisham Hospital	PD patients, *N* = 20
13. Lum et al. (29)	Canada	To describe PD patient and care partner perspectives on ACP to inform a patient- and care partner-centered framework for clinical care	University of Colorado, University of Alberta, and University of California San Francisco	PD patients, *N* = 30; caregivers, *N* = 30
14. Maffoni et al. (30)	Italy	To describe how people with Parkinson’s experience living with their condition over time	A 4-week multidisciplinary intensive rehabilitation treatment	PD patients, *N* = 27
15. Lennaerts-Kats et al. (31)	Dutch	To map the experiences of bereaved family caregivers during the period of informal care in the palliative care phase as well as after the death of their loved one with PD	Not mentioned	Caregivers, *N* = 10
16. Prizer et al. (32)	United States	To identify palliative needs of PD patients	Most frequently the participant’s home	PD patients, *N* = 23
17. Kurpershoek et al. (33)	United States	To explore the experiences, needs, and preferences of PD patients regarding the content and timing of ACP	At the outpatient clinic	PD patients, *N* = 20
18. Rosqvist et al. (34)	Sweden	To explore experiences of late-stage PD patients’ and their informal caregivers’ satisfaction with care and support, in order to better understand how they perceive the treatment and care they receive	Neurology departments and the municipality-based home healthcare and social services system	PD patients, *N* = 11; caregivers, *N* = 9
19. Churm et al. (35)	United Kingdom	To explore the views of people with PD and relatives on planning for the future and ACP	The participants’ home	PD patients and caregivers, *N* = 33
20. Kwok et al. (36)	China	To (a) understand the illness and adjustment experiences, (b) explore the reasons for psychological distress, and (c) discern the adjustment strategies adopted along the course of illness	At either the participant’s home or at patient support centers	PD patients, *N* = 14

**TABLE 2:** Methods and findings of included studies (*N* = 20).

References	Methods	Findings (themes)
1. Hudson et al. (18)	A qualitative descriptive study	Five themes: (1) emotional impact of diagnosis; (2) staying connected; (3) enduring financial hardship; (4) managing physical challenges; and (5) finding help for advanced stages.
2. Giles and Miyasaki (19)	A qualitative phenomenological study	Three main themes: (1) missing information; (2) being on your own; and (3) wanting and not wanting to know.
3. Hasson et al. (20)	An exploratory descriptive study	Four themes: (1) carers’ role and burden; (2) palliative care; (3) bereavement; and (4) access to health and social care services.
4. Tan et al. (21)	A qualitative exploratory study	Four themes: (1) coping and adaptation; (2) challenges of caregiving; (3) effects of caregiving on the caregivers; and (4) the need for better caregiver support.
5. Beaudet and Ducharme (22)	A qualitative descriptive study	Six themes: (1) meet the challenges associated with PD; (2) develop effective strategies for staying healthy; (3) solve new problems together; (4) access resources and plan for the future; (5) communicate better; and (6) fine tune roles.
6. Seamark et al. (23)	A qualitative study (not specific)	Three themes: (1) personal continuity; (2) informational continuity; and (3) organizational continuity of care.
7. van Rumund et al. (24)	A qualitative study (not specific)	Three themes: (1) emotional support and empathy; (2) organization of care; and (3) staff knowledge.
8. Murdock et al. (25)	A phenomenological qualitative study	Four themes: (1) physical; (2) psychological; (3) social; and (4) spiritual experiences.
9. Fox et al. (26)	A qualitative study (not specific)	Five themes: (1) high disease burden; (2) information and support needs: no “one-size-fits-all”; (3) crisis times needing extra support; (4) experience of healthcare services: feeling unsupported; and (5) experience and perceptions of palliative care.
10. Habermann and Shin (27)	A qualitative descriptive study	Three themes: (1) troublesome symptoms and problems; (2) unmet needs; and (3) concerns for the future.
11. Hurt et al. (28)	A qualitative study (not specific)	Six themes: (1) carer uncertainty about the patient’s symptoms and prognosis; (2) carer uncertainty about the patient’s medical management; (3) carer uncertainty about the patient’s self-management; (4) carer uncertainty about the patient’s impact; (5) carer uncertainty about the patient’s social functioning; and (6) carer uncertainty about themselves.
12. Hurt et al. (28)	A qualitative study (not specific)	Three main themes: (1) uncertainty about the relationship of non-motor symptoms to PD and lack of clarity around treatments were common; (2) embarrassment and communication difficulties were common for potentially sensitive symptoms such as sexual dysfunction; and (3) symptom perceptions and beliefs about help-seeking acted as barriers to reporting non-motor symptoms.
13. Lum et al. (29)	A qualitative descriptive study	Four themes: (1) personal definitions of ACP vary in the context of PD; (2) patient, relationship, and healthcare system barriers exist to engaging in ACP; (3) care partners play an active role in ACP; and 4) a palliative care approach positively influences ACP.
14. Maffoni et al. (30)	A grounded theory qualitative study	Three themes: (1) past; (2) present; and (3) future.
15. Lennaerts-Kats et al. (31)	A interpretative phenomenological study	Four themes: (1) “feeling like a ‘professional’ caregiver”; (2) “healthcare professionals do not always know what PD really means”; (3) “being on your own”; and (4) “being behind the times.”
16. Prizer et al. (32)	A qualitative study (not specific)	Two themes: (1) need for more effective care coordination; and (2) lack of healthcare education.
17. Kurpershoek et al. (33)	A qualitative study (not specific)	Two themes: (1) first, communication with various healthcare professionals about the diagnosis and ACP; and (2) communication about the uncertainty of the future disease burden.
18. Rosqvist et al. (34)	A qualitative study (not specific)	Five themes: (1) we are trying to get by both with and without the formal care; (2) dependence on others and scheduled days form everyday life; (3) there is a wish to get adequate help when it is needed; (4) mixed feelings on future housing and respite care; and (5) family responsibility and loyalty for a functioning everyday life.
19. Churm et al. (35)	A qualitative study (not specific)	Three themes: (1) awareness; (2) uncertaint; and (3) salience.
20. Kwok et al. (36)	A qualitative descriptive study	Two themes: (1) confronting the changes caused by PD; and (2) adjusting to living with PD.

PD, Parkinson’s disease; ACP, advanced care planning.

### 3.2 Experience and needs of palliative care

Based on a thematic analysis, four main themes emerged: (1) unmet emotional and informational needs; (2) needs for effective coordination of care; (3) planning for the future; and (4) symptom management.

#### 3.2.1 Theme 1: unmet emotional and informational needs

This theme is prominently addressed across the included literature (18–22, 24, 26–28, 31–36). Upon receiving a diagnosis of PD, patients and their family members often grapple with intense emotional turmoil, characterized by profound sadness and apprehensions about the future. Family caregivers describe the impact of the diagnosis with phrases such as “the bottom fell out of his world” and “he was broken-hearted” (18). This emotional upheaval is intricately linked to the unpredictable trajectory of the disease (19, 24, 26, 36).

However, when seeking information regarding prognosis, diagnosis, and home care services, patients and their families frequently encounter information gaps (19, 22, 26, 27, 33). Some are unsure about what questions to pose, while others are apprehensive about potential reprimands from physicians for making inquiries (19). Many patients report a sense of isolation due to a lack of sufficient information post-diagnosis. Some families turn to the internet for answers (19), and certain caregivers express a desire for more proactive and clear medical guidance (31, 34). Uncertainty emerges as a prevalent topic (28, 35), with both the unpredictability of the future and mixed messages regarding PD progression amplifying patient distress (20, 32).

Moreover, caregivers frequently note that their lifestyle is constrained, their roles have shifted, and they experience physical and emotional fatigue (21, 22, 28). They articulate an urgent need for enhanced caregiving support and more comprehensive guidance on PD management. Concurrently, both patients and caregivers indicate that their interactions with medical professionals often lack a sense of adequate support (32, 34). In summary, the unmet needs faced by PD patients and their families encompass a strong demand for emotional support and specialized information.

#### 3.2.2 Theme 2: needs for effective coordination of care

The theme of “needs for effective coordination of care” has been consistently emphasized in the selected literature. Most caregivers explicitly state their need for a more integrated care package, allowing them to regularly interact with experts and to be clearly directed to other types of services and information (20). This further underscores the notion that adopting a more integrated approach to PD services can enhance the effectiveness of the healthcare system (21). Continuity is a fundamental aspect of this theme. Family caregivers particularly value the consistent care from one or two primary caregivers or nurses, transitioning the relationship from strangers to trusted aides (23). However, frequent changes in nursing personnel are viewed negatively. The continuity of information becomes a focal point, with family caregivers having low expectations for information transfer between different care institutions. Many reported negative experiences related to information dissemination and communication (23). When the organizational aspect of care runs smoothly, family caregivers feel comforted and encouraged. Yet, many also reported negative facets of care organization, especially outside of working hours (23).

Budget constraints, frequent staff turnover, and time pressures are identified as challenges, all affecting the quality of care (24). This operational inefficiency leaves many feeling isolated, a sentiment exacerbated by limited interactions with the medical team. This often leads to a noticeable lack of ongoing support and service coherence (26). Communication between healthcare providers is a recurrent concern. Many participants feel that there is little communication among their doctors (32). Moreover, most feel that care is not well-integrated throughout the medical system, highlighting challenges in managing and coordinating care (32). Patients and caregivers express a preference for more frequent contact with PD specialists or neurologists. A common sentiment is that non-specialist medical institutions often lack adequate PD-specific knowledge, leading to mistakes, especially concerning medications (34). Many patients hope for more physiotherapy, emphasizing a shortage of specialized PD physiotherapy. Assistive devices and housing adaptations are deemed essential for daily living. In summary, there is a widespread desire among participants to receive appropriate assistance when needed. The capability and continuity of healthcare personnel are deemed crucial, especially in home healthcare. Challenges arising from staff turnover and communication errors are frequently lamented. There is a general hope for more flexibility and personalization in home healthcare (20).

#### 3.2.3 Theme 3: planning for the future

The theme of “planning for the future” pertains to how caregivers and patients anticipate and make plans for the progression of the disease and their perspectives on advance care planning (ACP). Most caregivers are pained by witnessing the physical and mental decline of patients. While they understand that death is inevitable, they still hope for the patient to remain at home for as long as possible (20, 34). Many Parkinson’s disease patients and their spouses strongly express the desire to stay in their homes for an extended duration. They are concerned about the lack of information that would help them make informed decisions for the future (27). Both patients and caregivers are keen to understand available resources, how to access them, and how to adequately plan for the future, emphasizing the importance of understanding and navigating available services (22).

Advance care planning is an emotionally charged topic, with varied opinions on when and how it should be approached. While some individuals remain hopeful about future treatments and do not wish to contemplate the end of life, others have given profound thought to care directives (26). Although many participants are not well-acquainted with or have misconceptions about palliative care (35), those who have experienced specialized palliative care hold it in high regard, recognizing its potential benefits in symptom control and support (26). In terms of communication around ACP, a majority of patients wish to discuss ACP with their healthcare providers to anticipate what the future may hold (33). While many have discussed issues related to ACP with their families (29), only a few have had such discussions with their medical teams. They express concerns about potential future symptoms, such as living in a wheelchair, becoming dependent, becoming a burden to their family, or having to reside in a nursing home (33). Many fear the onset of dementia and hope that discussions around ACP can address these uncertainties (33).

#### 3.2.4 Theme 4: symptom management

The theme of “symptom management” primarily focuses on how PD patients and caregivers confront, cope with, and manage the myriad symptoms of the disease. Symptom management encompasses various considerations. For instance, PD patients discuss a range of symptoms, like tremors, stiffness, and mobility issues, with a special focus on the common problems of “foot freezing” and movement difficulties (25). Medication is crucial in managing these symptoms, yet its “on/off” nature and side effects still have a negative impact on daily life (25). Also, the loss of speech and social interaction abilities, make it difficult for patients to communicate with others (18). Moreover, psychological and social experiences, such as sleep disorders, fatigue, depression, anxiety, stress, work, and recreational activities, are also pivotal aspects for PD patients to consider in symptom management (25, 37). In summary, PD patients face multifaceted challenges in symptom management. They have to deal not only with physical difficulties but also with psychological, social, and spiritual issues (30).

For caregivers, dealing with the patient’s increasingly severe symptoms poses a challenge. They frequently feel anxious and fearful, especially when the patient starts losing balance and falling. Family caregivers might also need to give up their employment to provide essential care, further exacerbating economic burdens (18). To better cope, they not only seek strength from their spiritual beliefs but also strive to maintain social activities (21). The moderate phase of PD presents a multitude of challenges for their caregivers, such as the loss of freedom, communication difficulties, and changes in roles and relationships. They emphasize the importance of finding ways to foster autonomy, hope, and learning (22, 30, 36).

## 4 Discussion

This scoping review investigated data from 20 qualitative studies to identify knowledge gaps in understanding the needs and experiences of individuals with PD who received palliative care. In our research findings, we discovered that unmet emotional and informational support is a recurring theme mentioned by the majority of PD patients and their caregivers. PD patients and their caregivers face numerous emotional challenges. As the disease progresses, many PD patients may experience feelings of depression, anxiety, loneliness, and low self-esteem (38). These emotional challenges can impact their daily quality of life, social interactions, and overall health (39). As such, emotional support becomes an urgent need for them. Moreover, changes in family dynamics, financial pressures, loss of patient autonomy, and feelings of social isolation also contribute to the caregivers’ burdens (40). Thus, offering them emotional support is equally vital. While doctors and medical teams are the primary sources of information, communities, support groups, patient associations, and online communities can also provide invaluable resources for PD patients and caregivers (41, 42). Additionally, mental health professionals and social workers can offer them emotional support (43, 44).

Parkinson’s disease progression is gradual, usually starting with minor tremors and escalating to other symptoms that affect daily life (45). Since there is currently no cure for PD and each patient’s response is relatively unique, it complicates the prediction of disease progression and prognosis (46). Given the uncertainty around the cause of PD, coupled with the variability in its progression and the complexity of its treatment, patients and caregivers might have misconceptions about the disease (47). They might struggle to interpret their symptoms, be unsure about selecting the best treatment options, or be unaware of how to handle potential complications. Therefore, providing disease-related informational support is of paramount importance. PD patients and caregivers need to understand how to manage symptoms, adjust daily activities to accommodate the disease’s limitations, and effectively communicate with doctors and medical teams (48). Moreover, advanced PD patients might need more medications and more frequent medical check-ups (49). Patients with PD often have comorbidities such as diabetes or hypertension due to age-related factors. Research (50) has found that PD shares common pathogenic mechanisms with diabetes in inflammation, oxidative stress, etc. Additionally, the use of antihypertensive drugs may sometimes exacerbate PD symptoms (51). Given these needs, medical institutions and relevant organizations should consider initiating PD educational programs or home visits to help patients and caregivers better understand and cope with the disease (52, 53).

Effective coordination of care is an urgent need for PD patients and their caregivers. van Vliet et al. (54) believed that there are barriers between specialist palliative care and neurology. Furthermore, a qualitative study in the Netherlands involving healthcare professionals on palliative care for PD patients showed that some interviewees supported the development of a PD palliative care system. However, they also felt the need to better understand the essence of palliative care. Some interviewees stated they would never introduce the term “palliative” and believed doctors should not discuss spiritual issues with patients, arguing that medical care should remain the core focus (55). This highlights that many medical professionals still lack a clear understanding of palliative care, often confusing it with end-of-life care. Hence, training for medical and care staff in palliative care and team building should be enhanced to improve the palliative care experience for advanced Parkinson’s patients and their caregivers.

As the disease progresses, PD patients might experience a gradual decline in physical and cognitive functions, leading to increased difficulties in daily life. This uncertain future leaves many PD patients and their caregivers feeling overwhelmed and anxious about how to prepare for what is ahead (49). Planning for the future can help PD patients and caregivers better cope with the challenges the disease presents and ensure their needs and wishes are respected and met (56). ACP provides PD patients and caregivers with a tool and opportunity to prepare for the future and ensure their medical wishes are respected. While it may be an emotional challenge (57), it offers assurance to PD patients and caregivers that their wishes will be respected and executed in critical moments.

In this study, the theme of symptom management provided us with an opportunity to deeply understand the numerous challenges faced by PD patients and their caregivers. The progression of Parkinson’s disease brings about a series of symptoms that profoundly affect the daily lives of those affected. For instance, the loss of language and social interaction capabilities often leads to feelings of isolation and depression (18). Many patients find themselves compelled to abandon their professional roles, leading to financial pressures. When caregivers, typically family members, have to make the tough decision to give up their jobs to cater to the escalating needs of the patient, this financial burden intensifies (18). Our study’s findings reveal that caregivers’ coping mechanisms are multifaceted: many find solace and strength from their spiritual beliefs, while others emphasize the importance of maintaining social connections and activities to alleviate feelings of isolation and helplessness (21). In conclusion, symptom management in PD is a complex, multi-dimensional task. Patients must combat the physical manifestations of the disease while also dealing with psychological, social, and spiritual challenges. It is heartening to see that, in the face of these adversities, both patients and caregivers proactively seek various strategies and resources (36). Medical professionals should acknowledge the positive coping strategies of patients and caregivers and offer necessary assistance to meet their needs in symptom management.

## 5 Limitations

This review had several limitations. We only included English publications and did not undertake a hand search of significant journals. We also did not include gray literature. Given all of these considerations, it could be possible that some data sources were overlooked. Although we included literature from various countries, the lack of data from Southeast Asian countries may be strongly related to cultural factors and suggests that future research should further explore the current state of palliative care services in these countries. Lastly, we did not perform a quality assessment of all included studies since the risk of bias/quality assessment was not often suggested in the retrieved reviews.

## 6 Conclusion

This scoping review revealed critical knowledge gaps regarding the needs and experiences of PD patients and their caregivers who receive palliative care. Our findings indicate that many PD patients and caregivers often face unmet emotional and informational needs, and they deeply aspire for effective care coordination. As they plan for the future, they try to prepare, even though some are not well-versed with ACP. Furthermore, they bear a significant burden in symptom management. While some patients and caregivers have found coping mechanisms, the majority still wrestle with numerous challenges on psychological, social, and spiritual fronts. In conclusion, further research and promotion of effective strategies and resources are essential to better address these needs and enhance their quality of life.

## Data availability statement

The original contributions presented in this study are included in the article/Supplementary material, further inquiries can be directed to the corresponding author.

## Author contributions

YL: Methodology, Resources, Writing – original draft. YTL: Conceptualization, Data curation, Formal analysis, Software, Writing – review & editing. YC: Conceptualization, Supervision, Writing – review & editing.
