# Supplementary material for: The palliative care needs and experiences of patients with advanced Parkinson’s disease: a qualitative scoping review
Source: Front Med (Lausanne). 2024 Apr 10;11:1362828. doi: 10.3389/fmed.2024.1362828 (PMC11039912; doi:10.3389/fmed.2024.1362828)
Supplement: Supplementary file 1 [file Table_1.DOCX]

Supplementary file 1 Search terms used for scoping review (PubMed, conducted in Sep 10^th^, n=579)

| Parkinson disease |
| --- |
| 1. Parkinson Disease[MeSH] OR Idiopathic Parkinson's Disease[Title/Abstract] OR Lewy Body Parkinson Disease[Title/Abstract] OR Lewy Body Parkinson's Disease[Title/Abstract] OR Primary Parkinsonism[Title/Abstract] OR Parkinsonism, Primary[Title/Abstract] OR Parkinson Disease, Idiopathic[Title/Abstract] OR Parkinson's Disease[Title/Abstract] OR Parkinson's Disease, Idiopathic[Title/Abstract] OR Parkinson's Disease, Lewy Body[Title/Abstract] OR Idiopathic Parkinson Disease[Title/Abstract] OR Paralysis Agitans[Title/Abstract] OR Parkinsonian[Title/Abstract] OR PD[Title/Abstract] OR Parkinson* [Title/Abstract] |
| Palliative care |
| 1. palliative care OR palliative medicine OR palliative treatment OR palliative therapy OR supportive care OR supportive medicine OR supportive treatment OR supportive therapy OR end of life care OR end of life medicine OR end of life treatment OR end of life therapy OR terminal care OR terminal medicine OR terminal treatment OR terminal therapy OR hospice care OR hospice medicine OR hospice treatment OR hospice therapy OR hospices[MeSH] |
| Qualitative study |
| 1. qualitative research[MeSH] OR qualitative research[Title/Abstract] OR focus groups[Title/Abstract] OR interviews[Title/Abstract] OR phenomenological[Title/Abstract] OR grounded theory[Title/Abstract] OR content analysis[Title/Abstract] OR narrative analysis[Title/Abstract] OR ethnographic[Title/Abstract] OR thematic analysis[Title/Abstract] |
| Needs or experiences |
| 1. “benefit*” OR “perception*” OR “feeling*” OR “perspective*” OR “experience*” OR “attitude*” OR “need*” |
